# Synthesis, Characterization, and Anti-Glioblastoma Activity of Andrographolide–Iron Oxide Nanoparticles (AG-IONPs)

**DOI:** 10.3390/biomedicines13102476

**Published:** 2025-10-11

**Authors:** Nanthini Ravi, Yazmin Bustami, Pandian Bothi Raja, Daruliza Kernain

**Affiliations:** 1 Institute for Research in Molecular Medicine, Universiti Sains Malaysia, Gelugor 11800, Pulau Pinang, Malaysia; nanthininans5@yahoo.com; 2 School of Biological Sciences, Universiti Sains Malaysia, Gelugor 11800, Pulau Pinang, Malaysia; ybustami@usm.my; 3 School of Chemical Sciences, Universiti Sains Malaysia, Gelugor 11800, Pulau Pinang, Malaysia; bothiraja@usm.my

**Keywords:** andrographolide, iron oxide nanoparticles, glioblastoma, magnetic nanoparticles, cytotoxicity, migration inhibition

## Abstract

**Background**: Glioblastoma multiforme (GBM) is an aggressive primary brain malignancy associated with poor prognosis and limited therapeutic options. Nanoparticle-based drug delivery systems provide a promising strategy to enhance treatment efficacy by circumventing barriers such as the blood–brain barrier. This study was conducted to synthesize, characterize, and evaluate the in vitro anticancer potential of andrographolide–iron oxide nanoparticles (AG-IONPs) against GBM cells. **Methods:** Iron oxide nanoparticles (IONPs) were synthesized through co-precipitation and subsequently functionalized with andrographolide. Morphology, size, and surface charge were assessed by transmission electron microscopy (TEM), dynamic light scattering (DLS), and zeta potential analysis. Functionalization was confirmed by Fourier-transform infrared spectroscopy (FTIR) and UV–Vis spectroscopy. Nanoparticle stability was monitored over three months. Cytotoxicity toward DBTRG-05MG cells was evaluated using MTT assays at 24, 48, and 72 h, while anti-migratory effects were determined using scratch-wound assays. Results: TEM analysis revealed nearly spherical IONPs (7.0 ± 0.15 nm) and AG-IONPs (13.5 ± 1.25 nm). DLS indicated an increased hydrodynamic diameter following functionalization, while zeta potential values decreased from +21.22 ± 1.58 mV to +8.68 ± 0.87 mV. The successful incorporation of andrographolide was confirmed by FTIR and UV–Vis spectra. AG-IONPs demonstrated excellent colloidal stability for up to three months. Cytotoxicity assays revealed a dose- and time-dependent decrease in cell viability, with LC_50_ values declining from 44.01 ± 3.23 μM (24 h) to 15.82 ± 2.30 μM (72 h). Scratch-wound assays further showed significant inhibition of cell migration relative to untreated controls. **Conclusions:** AG-IONPs exhibit favorable physicochemical properties, long-term stability, and potent anti-proliferative and anti-migratory effects against GBM cells in vitro. These findings support their potential as a multifunctional therapeutic platform, warranting further preclinical investigation.

## 1. Introduction

Glioblastoma Multiforme (GBM) is the most aggressive and prevalent malignant brain tumor in adults [1]. Despite maximal surgical resection in combination with radiotherapy and temozolomide, patient prognosis remains poor, with median survival scarcely exceeding two years and a five-year survival rate below 10% [2]. This dismal outcome is largely attributed to extensive tumor infiltration, pronounced genetic heterogeneity, and intrinsic resistance to cytotoxic therapies, which inevitably result in recurrence [3]. A critical barrier to therapeutic efficacy is the blood–brain barrier (BBB), a highly selective interface that prevents the majority of large molecules and many small-molecule drugs from reaching sufficient concentrations at the tumor site [4]. Nanotechnology-based drug delivery systems have therefore attracted increasing attention as promising strategies to address these challenges [5]. Nanoparticles can be engineered to enhance drug solubility, extend systemic circulation, enable controlled release, and exploit both active and passive targeting mechanisms to cross the BBB [6]. Among these approaches, iron oxide nanoparticles (IONPs) are particularly advantageous in neuro-oncology due to their biocompatibility, magnetic responsiveness, and dual diagnostic–therapeutic potential [7]. Such properties render IONPs effective multifunctional carriers, improving delivery precision while minimizing off-target toxicity. Andrographolide (AG), a labdane diterpenoid isolated from *Andrographis paniculata*, has demonstrated notable anticancer properties, including the induction of apoptosis, inhibition of proliferation, and suppression of metastasis [8]. Its high lipid solubility enables BBB penetration [9]; however, clinical application remains limited due to poor aqueous solubility, low bioavailability, and limited stability [10]. Conjugation with IONPs represents a rational strategy to overcome these limitations, offering improved solubility, enhanced stability, and targeted delivery to GBM tissues. This study was designed to synthesize and characterize andrographolide-loaded iron oxide nanoparticles (AG-IONPs) and to evaluate their potential as a nanocarrier system for glioblastoma therapy by combining the therapeutic efficacy of AG with the delivery advantages of IONPs.

## 2. Materials and Methods

### 2.1. Synthesis of Iron Oxide Nanoparticles (IONPs)

Iron oxide nanoparticles (IONPs) were synthesized via a modified reverse co-precipitation method [11]. Briefly, 50 mL of 1 M sodium hydroxide solution and 1 mL of 1 mM trisodium citrate were mixed with 50 mL of deionized water and stirred for 3 min using a magnetic stirrer, (YX Magnetic, Sierre, Switzerland). Ferrous sulfate heptahydrate (FeSO_4_·7H_2_O; 55.6 mg) was subsequently added, and the mixture was stirred vigorously for 10 min at room temperature. The appearance of a brownish precipitate indicated nanoparticle formation.The suspension was subjected to microwave irradiation for 1 min and then allowed to cool overnight. Nanoparticles were collected using a permanent magnet, (YX Magnetic, Sierre, Switzerland) and the supernatant was discarded. The pellet was air-dried overnight before being treated with 10 mL of 6 M nitric acid and stirred for 10 min at room temperature, yielding a brown solution. This mixture was centrifuged at 6500 rpm for 10 min, and the pellet was resuspended in 10 mL of deionized water, followed by magnetic separation overnight. The final IONP pellet was dissolved in 10 mL of deionized water and stored at 4 °C until further use.

### 2.2. Synthesis of Andrographolide-Loaded Iron Oxide Nanoparticles (AG-IONPs)

AG-IONPs were prepared by mixing 500 μL of 1 M andrographolide (AG) solution with 1 mL of IONPs, followed by vortexing for 1 min. The mixture was subjected to sonication for a total of 15 min, applied in 5 min intervals and repeated seven times. The resulting precipitate was magnetically separated overnight at 4 °C. The supernatant was discarded, and the pellet was resuspended in 5 mL of deionized water and stored at 4 °C until further use.

### 2.3. Determination of Encapsulation and Loading Efficiency

Encapsulation efficiency (EE) and loading efficiency (LE) were determined as previously described, with minor modifications. Briefly, 5 mL of AG-IONPs were purified by ultracentrifugation (18,000 rpm, 75 min, 4 °C), and the concentration of unencapsulated AG in the supernatant was quantified spectrophotometrically at 284 nm. A standard calibration curve was prepared using serial dilutions of AG. The EE and LE were calculated using the following equations:

(1) $$\mathrm{EE}\%=\frac{\mathrm{Total}\;\mathrm{Drug}-\mathrm{Free}\;\mathrm{Drug}}{\mathrm{Total}\;\mathrm{Drug}}\times 100$$

(2) $$\mathrm{LE}\%=\frac{\mathrm{Amount}\;\mathrm{of}\;\mathrm{drug}\;\mathrm{in}\;\mathrm{nanoparticles}}{\mathrm{Amount}\;\mathrm{of}\;\mathrm{nanoparticles}}\times 100$$

### 2.4. Characterization of Nanoparticles

#### 2.4.1. UV–Visible Spectroscopy

UV–Vis spectra of IONPs and AG-IONPs were recorded using a UV-1800 spectrophotometer (Shimadzu, Kyoto, Japan) across the wavelength range of 300–800 nm.

#### 2.4.2. Transmission Electron Microscopy (TEM)

Morphology and particle size were examined using a Philips CM12 TEM (Electron Microscopy Unit, School of Biological Sciences, Universiti Sains Malaysia, George Town, Malaysia). A drop of filtered nanoparticle suspension was deposited onto a copper grid and air-dried for 30 min. Particle size was analyzed using Docu Version 3.2 image analysis software.

#### 2.4.3. Dynamic Light Scattering (DLS) and Zeta Potential

Hydrodynamic diameter and zeta potential were measured using a Malvern Zetasizer (MalvernPanalytical Inc., Malvern, Worcestershire, UK). Samples were filtered through a 0.20 μm PES syringe filter, dispersed in deionized water, and sonicated for 15 min prior to measurement.

#### 2.4.4. Fourier-Transform Infrared Spectroscopy (FTIR)

Functional groups were analyzed using attenuated total reflectance–FTIR (ATR-FTIR; Thermo Nicolet IR 200, Madison, WI, USA in the range of 400–4000 cm^−1^.

### 2.5. Stability Studies

The colloidal stability of IONPs and AG-IONPs was assessed by monitoring UV–Vis absorbance peaks (285 nm for IONPs and 352 nm for AG-IONPs) at defined storage intervals (24 h, 1 week, 1 month, 2 months, and 3 months) at 4 °C.

### 2.6. Cell Culture and Cytotoxicity Assay

DBTRG-05MG human glioblastoma cells (CRL-2020, ATCC, Manassas, VA, USA) were maintained in RPMI-1640 medium supplemented with 10% fetal bovine serum (FBS) and 1% penicillin–streptomycin at 37 °C in a humidified incubator containing 5% CO_2_. For cytotoxicity analysis, cells were seeded at a density of 5000 cells/well in 96-well plates and incubated overnight. AG-IONPs or temozolomide (TMZ) were added at concentrations ranging from 0.7813 μM to 200 μM, and cells were incubated for 24, 48, or 72 h. WST-1 reagent (10 μL) was then added to each well and incubated for 45 min. Absorbance was measured at 430 nm with a reference wavelength of 630 nm using a microplate reader (SpectraMax, San Jose, CA, USA).

### 2.7. Scratch Assay

DBTRG-05MG cells were seeded at a density of 3.5 × 10^5^ cells/well in 6-well plates and cultured until 80–90% confluence. A linear scratch was made using a sterile 1000 μL pipette tip, and detached cells were removed by PBS washing. Fresh medium containing AG-IONPs at the LC_50_ concentration (15.82 μM) was added. Images of wound closure were captured at 0, 24, 48, and 72 h using an inverted microscope. The percentage of wound closure was quantified using ImageJ software (Biotek, Agilent, Santa Clara, CA, USA) according to the following equation:

(3) $$Migration\;rate\%=\left[\frac{\left(A_{t=0h}-A_{t=∆h}\right)}{\left(A_{t=0h}\right)}\right]\times 100\%$$

*A_t_*_=0h_ is the area of the wound measured immediately after scratching (*t* = 0 h).
*A_t_*_=∆h_ is the area of the wound measured h hours after the scratch is performed.

### 2.8. Apoptosis Assay

DBTRG cell lines were treated with IONPs and AG-IONPs with the concentration of 15.82 μM for 24 h and 48 before the apoptosis assay is carried out. The cells are then centrifuged to obtain cell pellet which is resuspended with 1× Annexin Binding Buffer (AVBB). The cell suspension is transferred to round bottom culture tube followed by the addition of 5 μL of Annexin V-Fitc and 5 μL of PI staining in the tube. The tubes are gently mixed followed by the incubation in the dark at room temperature for 15 min. Then, the AVBB is added into the tubes to stop the reaction and kept on ice followed by the apoptosis analysis using flow cytometer, SpectraMax, San Jose, CA, USA.

## 3. Results

### 3.1. Synthesis and Visual Confirmation

**Figure 1:**
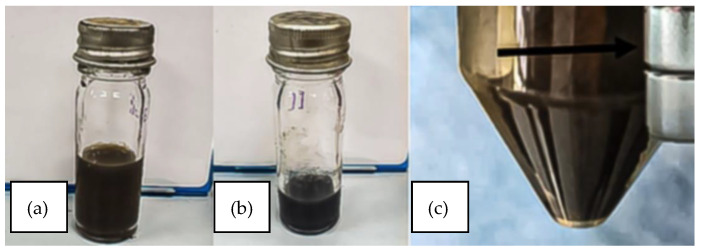
(**a**) Aqueous suspension of synthesized iron oxide nanoparticles (IONPs) exhibiting a brownish coloration, indicative of nanoparticle formation. (**b**) Andrographolide-functionalized IONPs (AG-IONPs) displaying a visible color shift compared to bare IONPs, confirming surface modification with andrographolide. (**c**) Magnetic attraction of IONPs toward an external permanent magnet, demonstrating their superparamagnetic behavior and suitability for magnetically guided drug delivery applications.

The reverse co-precipitation method enabled the rapid synthesis of IONPs, as indicated by the immediate appearance of brownish precipitates characteristic of iron oxide formation. Their magnetic attraction to an external magnet confirmed the particles’ superparamagnetic behavior, consistent with superparamagnetic iron oxide nanoparticles (SPIONs). Following conjugation with andrographolide (AG), the suspension underwent a visible color change from brown to brownish-black, suggesting successful surface functionalization (Figure 1). This alteration in optical appearance is consistent with organic ligand binding to nanoparticle surfaces, which modifies their light absorption properties.

### 3.2. UV–Visible Spectroscopy

**Figure 2:**
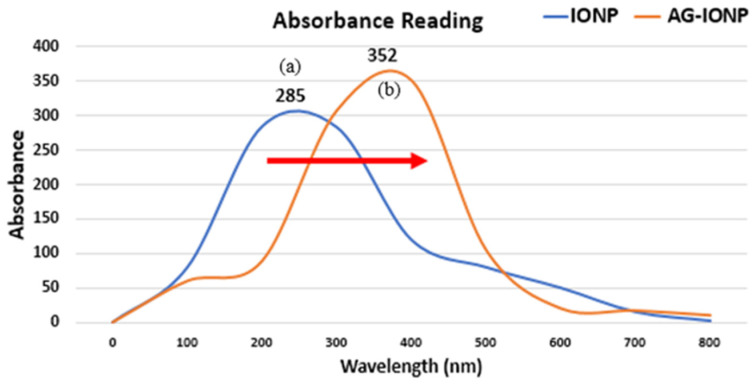
UV–visible absorbance spectra of iron oxide nanoparticles (IONPs) and andrographolide-loaded iron oxide nanoparticles (AG-IONPs). IONPs exhibited a distinct absorption peak at 285 nm (**a**), while AG-IONPs showed a red-shifted peak at 352 nm (**b**).

In Figure 2, The absorption spectrum of bare IONPs exhibited a peak at ~285 nm, attributed to Fe–O charge transfer transitions [12]. After AG loading, the peak shifted to ~352 nm, indicating a red shift. This shift may result from (1) increased particle size after AG coating, which alters the surface plasmon resonance, and (2) changes in the dielectric constant of the surrounding medium due to AG’s presence. A red shift is often used as an early, indirect indicator of successful surface functionalization in nanomaterial synthesis [13].

### 3.3. Encapsulation and Loading Efficiency

AG-IONPs demonstrated an encapsulation efficiency (EE) of 60% and a loading efficiency (LE) of 42.85%. For a hydrophobic compound like AG, these values are relatively high, indicating that the nanoparticle matrix provides a favorable environment for AG incorporation. High EE is critical for maximizing therapeutic payload, while a reasonable LE ensures efficient delivery without excessive carrier material, which could dilute drug concentration at the target site.

### 3.4. Size and Morphology (TEM and DLS)

**Figure 3:**
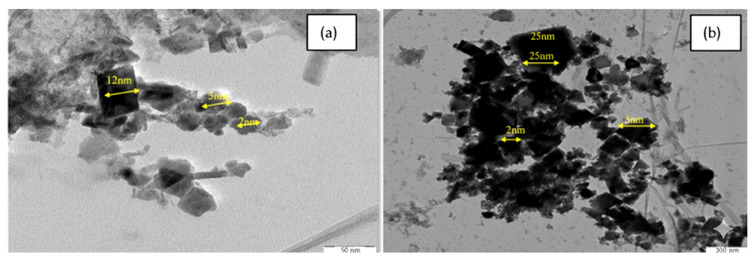
Transmission electron microscopy (TEM) images of (**a**) iron oxide nanoparticles (IONPs) and (**b**) andrographolide-loaded iron oxide nanoparticles (AG-IONPs). IONPs appeared as nearly spherical particles with uniform morphology, whereas AG-IONPs exhibited slightly larger sizes following surface functionalization with andrographolide.

TEM images revealed well-dispersed, quasi-spheroidal nanoparticles, with IONPs averaging 7.0 ± 0.15 nm and AG-IONPs averaging 13.5 ± 1.25 nm (Figure 3). The observed increase in size confirms successful andrographolide (AG) coating. DLS measurements showed slightly larger hydrodynamic diameters (2–12 nm for IONPs and 2–25 nm for AG-IONPs), attributed to the solvation layer surrounding the particles in suspension. The overall size range of 10–25 nm falls within the optimal window for biomedical applications. The nanoparticles demonstrated a narrow size distribution, with diameters spanning approximately 2–25 nm, and an average particle diameter of 9.8 ± 3.5 nm (mean ± SD), indicating uniform morphology and good monodispersity.

Average particle size was determined using transmission electron microscopy (TEM) and dynamic light scattering (DLS), while surface charge was assessed by zeta potential analysis. Data highlight the increase in hydrodynamic diameter and reduction in surface charge following andrographolide (AG) functionalization of iron oxide nanoparticles (IONPs), confirming successful surface modification (Table 1).

**Figure 4:**
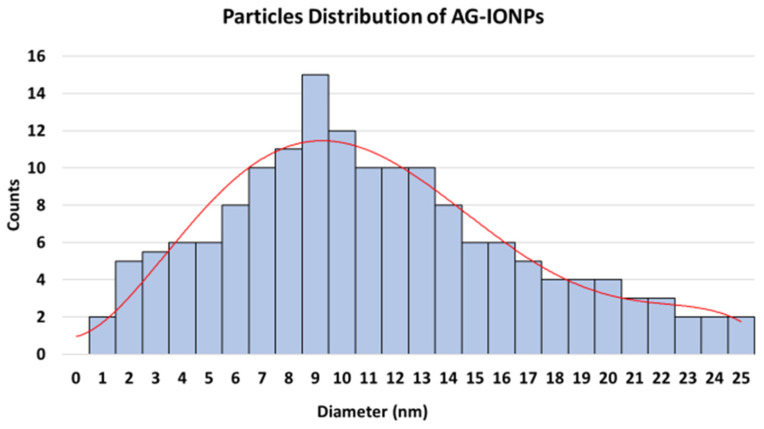
Particle size distribution of andrographolide-loaded iron oxide nanoparticles (AG-IONPs) obtained from transmission electron microscopy (TEM) image analysis.

The histogram (Figure 4) demonstrates a relatively narrow size distribution, with an average particle diameter of approximately 13.5 ± 1.25 nm, confirming uniformity following surface functionalization with andrographolide.

### 3.5. Zeta Potential

**Figure 5:**
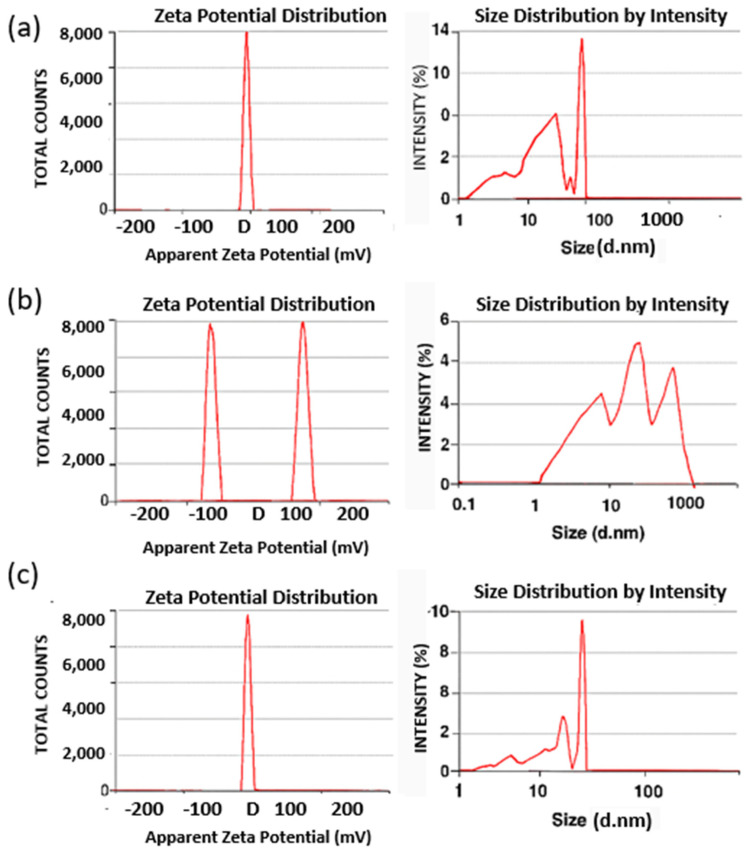
Dynamic light scattering (DLS) and zeta potential analysis of nanoparticles. (**a**) Iron oxide nanoparticles (IONPs) displayed a narrow size distribution and a sharp zeta potential peak (+21.22 ± 1.58 mV), confirming good colloidal stability. (**b**) Free andrographolide (AG) showed a polydisperse size profile with multiple zeta potential peaks, indicating instability and a tendency toward aggregation in aqueous suspension. (**c**) Andrographolide-loaded iron oxide nanoparticles (AG-IONPs) exhibited a narrower size distribution and a reduced zeta potential (+8.68 ± 0.87 mV), reflecting successful surface functionalization with AG and improved dispersion stability compared with free AG.

The zeta potential decreased from +21.22 mV for IONPs to +8.68 mV for AG-IONPs following conjugation with andrographolide (AG) (Figure 5). Bare IONPs exhibited a high positive surface charge due to protonation of surface hydroxyl groups in aqueous media. In contrast, AG contains neutral and weakly polar functional groups, which reduced the net surface charge upon coating. Although the zeta potential value decreased, +8.68 mV still reflects moderate colloidal stability, and stability tests confirmed that no aggregation occurred over a three-month storage period.

### 3.6. FTIR Confirmation of Surface Functionalization

**Figure 6:**
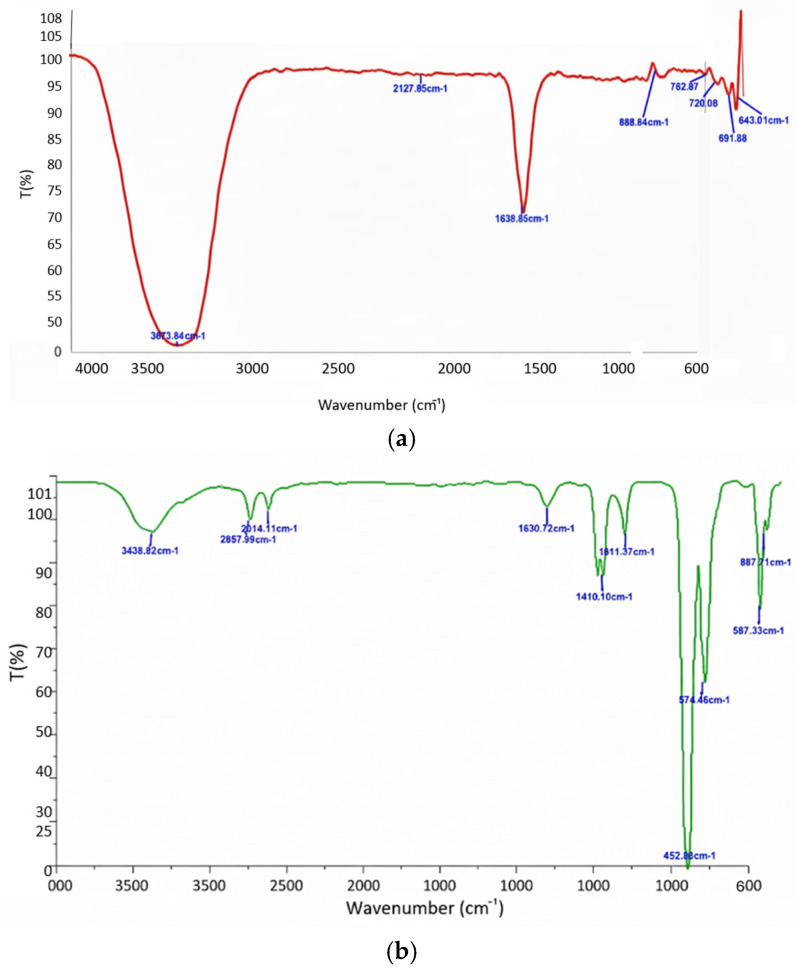
ATR-FTIR spectra of (**a**) bare IONPs and (**b**) andrographolide-coated IONPs (AG-IONPs). Both samples exhibited a characteristic Fe–O stretching vibration at ~640 cm^−1^, confirming retention of the iron oxide core structure. AG-IONPs showed additional absorption bands at ~3438 cm^−1^ (O–H stretching), ~1658 cm^−1^ (H–O–H bending of adsorbed water), and ~1023 cm^−1^ (C–H skeletal deformations), consistent with andrographolide’s functional groups. The presence of these peaks confirms successful surface functionalization of IONPs with AG, most likely via physical adsorption and hydrogen bonding interactions.

ATR-FTIR analysis (Figure 6) revealed a characteristic Fe–O stretching vibration (~640 cm^−1^) in both bare and AG-coated IONPs, confirming that the iron oxide core structure remained intact. In AG-IONPs, additional absorption peaks were observed at ~3438 cm^−1^ (O–H stretching), ~1658 cm^−1^ (H–O–H bending vibration), and ~1023 cm^−1^ (C–H skeletal deformations), consistent with the chemical structure of andrographolide. These spectral features verify the successful presence of AG on the nanoparticle surface, most likely through physical adsorption and hydrogen bonding interactions.

### 3.7. Stability Studies

**Figure 7:**
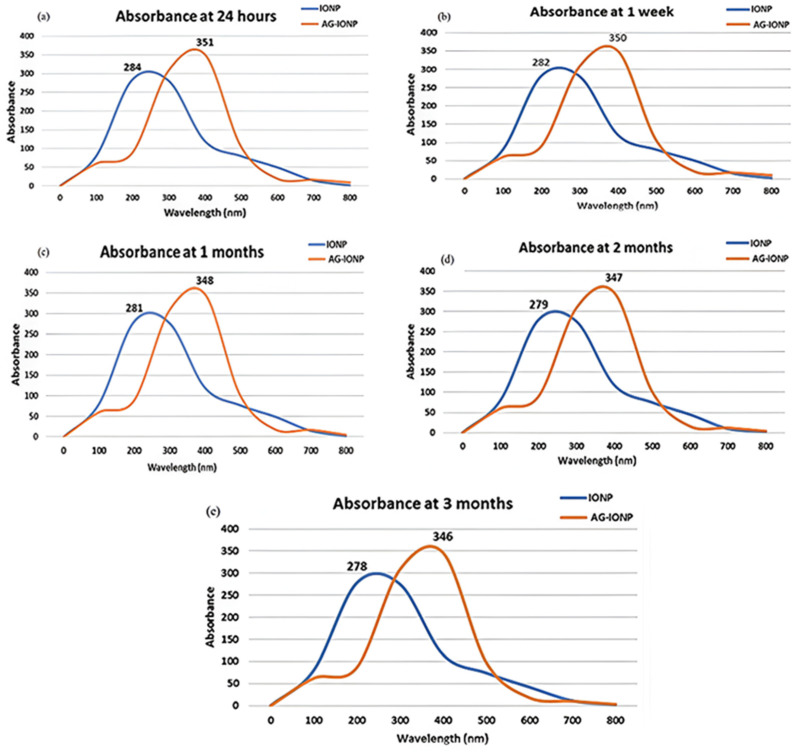
UV–visible absorbance spectra of iron oxide nanoparticles (IONPs) and andrographolide-loaded iron oxide nanoparticles (AG-IONPs) monitored over different storage intervals: (**a**) 24 h, (**b**) 1 week, (**c**) 1 month, (**d**) 2 months, and (**e**) 3 months. IONPs maintained a characteristic peak at ~285 nm, while AG-IONPs consistently exhibited a red-shifted peak at ~352 nm, confirming the stability of andrographolide functionalization. Minimal changes in absorbance intensity or peak position across the three-month period indicate excellent colloidal stability and long-term preservation of nanoparticle integrity.

UV–Vis monitoring over a 3-month period showed only minor peak shifts (≤7 nm) for both IONPs and AG-IONPs (Figure 7). Slight narrowing of the peaks may reflect minor particle rearrangements but does not indicate significant aggregation. Maintaining stability during storage is essential for nanomedicines to ensure reproducible therapeutic efficacy and extended shelf-life.

### 3.8. Cytotoxicity (WST-1 Assay)

**Figure 8:**
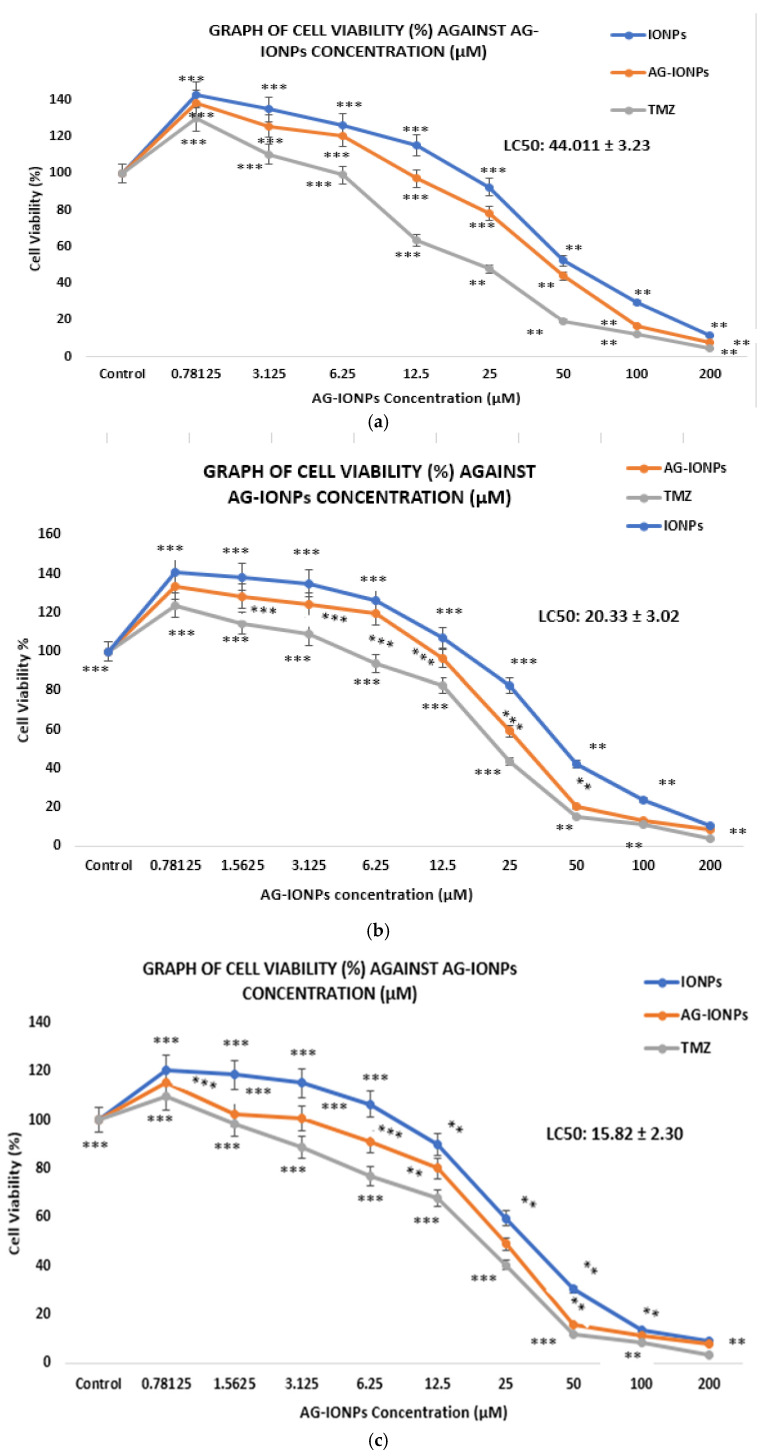
Cell viability of DBTRG-05MG glioblastoma cells following treatment with iron oxide nanoparticles (IONPs), andrographolide-loaded IONPs (AG-IONPs), and temozolomide (TMZ) at various concentrations after (**a**) 24 h, (**b**) 48 h, and (**c**) 72 h. AG-IONPs demonstrated a dose- and time-dependent reduction in cell viability, with significantly greater cytotoxicity compared with IONPs alone. The effects were comparable to those of TMZ, the standard chemotherapeutic control, indicating the potential of AG-IONPs as an effective therapeutic candidate. Data are expressed as mean ± standard deviation (SD). Statistical significance was determined using one-way ANOVA with post hoc Tukey test ** *p* < 0.01, *** *p* < 0.0001 versus control.

AG-IONPs exhibited a clear dose- and time-dependent cytotoxic effect on DBTRG-05MG glioblastoma cells (Figure 8). The LC_50_ values decreased significantly from 44.01 μM at 24 h to 15.82 μM at 72 h (Table 2), indicating enhanced cell-killing efficiency with prolonged exposure. This pattern suggests either a sustained release of andrographolide from the nanoparticle matrix or gradual intracellular accumulation. Temozolomide (TMZ), the standard chemotherapeutic for GBM, showed lower LC_50_ values across all time points, as expected for a clinically optimized small molecule. Nevertheless, the cytotoxic activity of AG-IONPs is noteworthy for a natural product-based Nano formulation, particularly one that may offer reduced systemic toxicity compared with TMZ.

### 3.9. Migration Inhibition (Scratch Assay)

**Figure 9:**
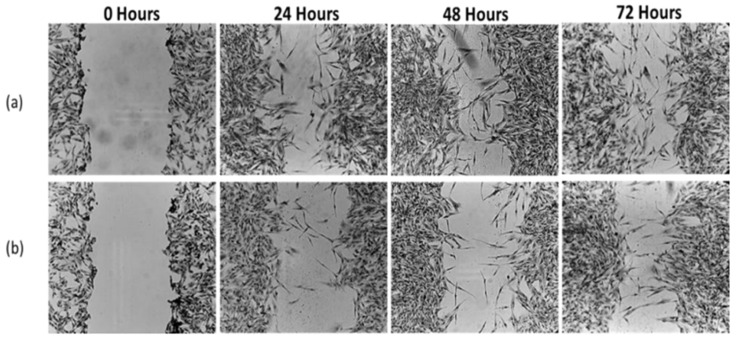
Migration rate of DBTRG-05MG glioblastoma cells treated with andrographolide-loaded iron oxide nanoparticles (AG-IONPs) compared with untreated controls. Cells were exposed to 15.82 μM AG-IONPs (LC_50_ concentration) or complete medium alone for 24, 48, and 72 h. AG-IONP treatment (**b**) markedly inhibited cell migration in a time-dependent manner compared with the control (**a**), as evidenced by slower wound closure in scratch assays. These findings demonstrate the anti-migratory potential of AG-IONPs in addition to their cytotoxic effects.

Control DBTRG-05MG cells exhibited progressive wound closure over 72 h, reflecting active migratory capacity in Figure 9. In contrast, AG-IONP–treated cells showed markedly reduced migration, with only ~47% closure at 72 h compared with near-complete closure in controls. These findings indicate that AG-IONPs effectively impair glioblastoma cell motility, a key process driving tumor invasion and recurrence. The known anti-migratory activity of andrographolide, mediated in part through inhibition of ERK1/2-dependent matrix metalloproteinase expression, may be further enhanced by nanoparticle-based delivery, resulting in more sustained intracellular activity.

**Figure 10:**
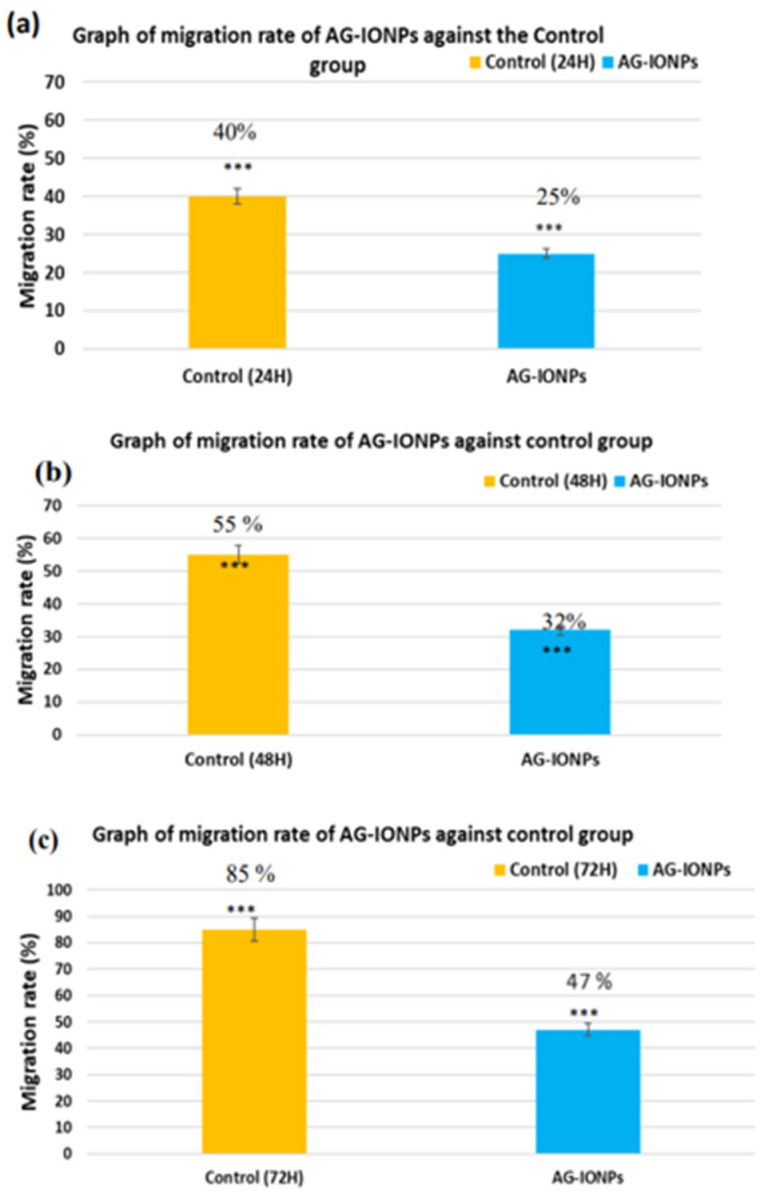
Scratch-wound assay showing the migration rate of DBTRG-05MG glioblastoma cells treated with andrographolide-loaded iron oxide nanoparticles (AG-IONPs) compared with untreated controls. Cells were exposed to 15.82 μM AG-IONPs (LC_50_ concentration) or complete medium alone for 24, 48, and 72 h. Data are presented as mean ± standard deviation (SD). Statistical analysis was performed using one-way ANOVA followed by Tukey’s post hoc test; *** *p* < 0.001 versus control.

In control wells, wound closure progressed steadily, with near-complete closure by 72 h, whereas AG-IONP treatment significantly suppressed migration in a time-dependent manner, with wounds remaining largely open. These results in Figure 10, confirm the anti-migratory effect of AG-IONPs, suggesting potential to limit glioblastoma invasiveness in addition to reducing cell viability.

### 3.10. Apoptotic Effects of IONPs and AG-IONPs on Glioblastoma

**Figure 11:**
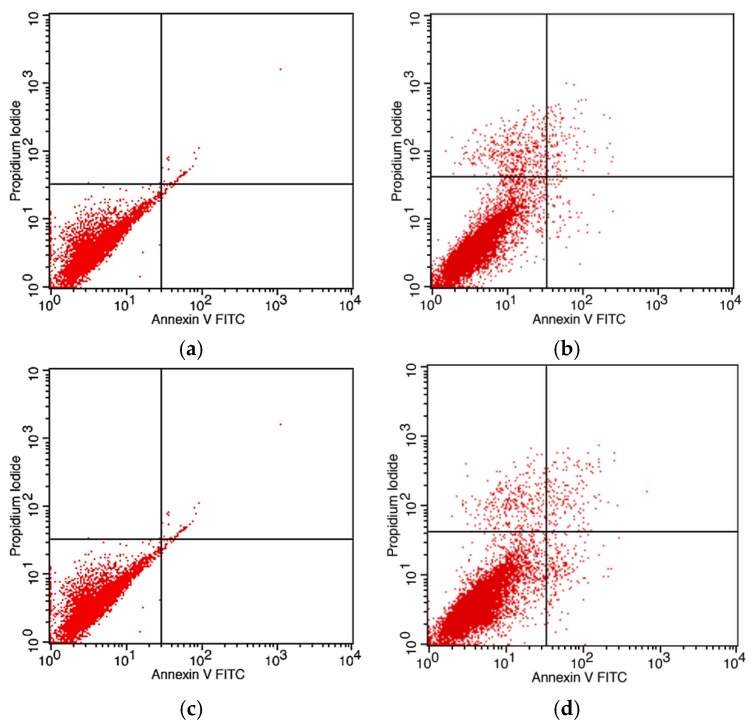
Apoptosis analysis of DBTRG-05MG glioblastoma cells following treatment with IONPs and AG-IONPs. DBTRG-05MG cells were treated with bare iron oxide nanoparticles (IONPs) (**a**,**c**) or andrographolide-conjugated IONPs (AG-IONPs) (**b**,**d**) for 24 h (**a**,**b**) and 48 h (**c**,**d**). Apoptosis was assessed by Annexin V-FITC/PI staining and flow cytometry. Bare IONPs induced negligible apoptosis, whereas AG-IONPs significantly increased early and late apoptotic populations. Apoptotic activity was more pronounced after 48 h compared with 24 h. Data represent mean ± SD from three independent experiments, with significance determined by one-way ANOVA (<0.05).

Apoptosis was evaluated in DBTRG-05MG glioblastoma cells following treatment with bare IONPs and AG-IONPs for 24 h and 48 h using Annexin V-FITC/PI staining and flow cytometry. Cells treated with bare IONPs (Figure 11a,c) showed negligible apoptosis, with most events clustering in the viable cell quadrant. In contrast, AG-IONPs (Figure 11b,d) induced a marked increase in both early and late apoptotic populations compared with untreated controls. The effect was time-dependent, with apoptosis more pronounced at 48 h than at 24 h, demonstrating that AG conjugation significantly enhances the pro-apoptotic activity of IONPs.

## 4. Discussion

Glioblastoma multiforme (GBM) remains one of the most lethal brain malignancies, characterized by rapid proliferation, extensive infiltration, and resistance to conventional therapies. Despite advances in surgery, radiotherapy, and chemotherapy, the median survival of patients rarely exceeds 15 months [14,15]. A key obstacle in effective treatment is the blood–brain barrier (BBB), which severely limits the penetration of chemotherapeutic drugs into tumor tissues [16]. Nanotechnology-based drug delivery systems offer a promising solution by improving solubility, prolonging drug release, and enabling targeted delivery.

In this study, andrographolide (AG) was selected as the therapeutic candidate due to its established anticancer properties, including induction of apoptosis, inhibition of cell proliferation, and suppression of migration [17]. However, its clinical use is constrained by poor aqueous solubility, low oral bioavailability, and rapid systemic clearance [18]. Encapsulation within iron oxide nanoparticles (IONPs) was therefore pursued to enhance drug stability, facilitate magnetic targeting, and enable potential image-guided applications [19].

The reverse co-precipitation method produced well-dispersed, quasi-spherical IONPs with an average diameter of 7.0 nm, which increased to 13.5 nm following AG incorporation. Such dimensions are considered favorable for biomedical applications, as they are sufficiently small to cross tumor vasculature but large enough to avoid rapid renal clearance [20]. TEM and DLS analyses confirmed the increase in hydrodynamic size after AG attachment, in agreement with earlier reports that small-molecule surface modification can enlarge nanoparticle size and cause a red shift in UV–Vis absorption [21]. In this study, the red shift from 285 nm to 352 nm likely reflects changes in the local dielectric environment induced by AG’s organic groups, coupled with possible plasmon coupling effects [22].

Encapsulation efficiency (EE) and loading efficiency (LE) are critical for therapeutic success. For hydrophobic natural compounds such as AG, achieving an EE of 60% and LE of 42.85% is remarkable, with values comparable to other effective nanoparticle-based systems [23]. These efficiencies are especially important for GBM treatment, where even modest increases in local drug concentration may significantly enhance therapeutic outcomes given the restrictive nature of the BBB [24].

Surface charge analysis revealed a reduction in zeta potential from +21.22 mV in bare IONPs to +8.68 mV in AG-IONPs. Although this reduction could theoretically lower electrostatic repulsion and increase aggregation, stability tests conducted over three months showed no evidence of significant particle aggregation. This stability may be attributed to combined electrostatic and steric stabilization effects provided by AG molecules on the nanoparticle surface [25]. Importantly, AG-IONPs maintained colloidal stability for at least three months at 4 °C, underscoring their robustness for storage and scalability [26].

FTIR spectra further confirmed AG surface modification. In addition to Fe–O stretching peaks observed in both coated and uncoated IONPs, AG-IONPs displayed distinctive absorption bands at ~3438 cm^−1^ (O–H stretching), 1023 cm^−1^ (C–H skeletal vibrations), and 1658 cm^−1^ (water bending). These align with AG’s chemical structure, indicating successful surface attachment, most likely mediated by hydrogen bonding and other non-covalent interactions [27]. The preservation of both Fe–O bonds and AG’s functional groups suggests that conjugation maintained the structural integrity of the IONP core while retaining AG’s bioactivity [28].

Cytotoxicity studies demonstrated that bare IONPs exerted minimal effects on DBTRG-05MG cells, consistent with reports of their biocompatibility at low concentrations [29]. By contrast, AG-IONPs showed a clear dose- and time-dependent reduction in cell viability, with LC_50_ values decreasing from 44.01 μM at 24 h to 15.82 μM at 72 h. This trend suggests sustained AG release and or progressive intracellular accumulation. While temozolomide (TMZ) displayed lower LC_50_ values, direct comparisons are limited, as nanoparticle formulations differ fundamentally from conventional small molecules in pharmacokinetics, biodistribution, and toxicity profiles [30].

Beyond cytotoxicity, GBM’s invasive nature plays a central role in recurrence and poor prognosis. Our scratch-wound assay revealed that AG-IONPs substantially impaired cell migration, reducing wound closure to ~47% at 72 h compared to near-complete closure in controls. This observation is consistent with reports that AG inhibits migration through ERK1/2 modulation and suppression of MMP expression [31]. Although molecular confirmation was not performed in this study, the observed anti-migratory effect supports AG’s potential activity when delivered via nanoparticle formulations.

Several limitations must be acknowledged. First, all experiments were performed on a single glioblastoma cell line (DBTRG-05MG). Given GBM heterogeneity, validation across additional lines and patient-derived cultures is necessary, alongside selectivity testing in non-cancerous neural cells. Second, the WST-1 assay evaluates overall metabolic activity but cannot distinguish between apoptotic and necrotic cell death. Incorporating apoptosis-specific assays such as Annexin V/PI staining would yield more detailed mechanistic insights. Third, nanoparticle stability was assessed only under aqueous storage; stability in biological fluids, where protein corona formation and ionic interactions may alter particle properties, remains untested [32].

Another limitation is the absence of direct evidence for BBB penetration. Although AG is lipophilic and iron oxide nanoparticles have been reported to cross the BBB under certain conditions, this was not explored in this study. Future work should employ in vitro BBB models and in vivo pharmacokinetic analyses to verify brain-targeting efficiency. Furthermore, biodistribution studies are essential to assess accumulation in clearance organs such as the liver, spleen, and kidneys, as this is a common challenge for iron oxide-based nanocarriers [33].

Despite these limitations, the study results provide a strong basis for further development of AG-IONPs as a multifunctional therapeutic platform. Their dual cytotoxic and anti-migratory activities indicate potential to address multiple hallmarks of GBM progression. Future studies should investigate synergistic effects by combining AG-IONPs with standard therapies, including TMZ and radiotherapy, as multimodal regimens remain central to GBM treatment. Incorporation into hybrid nanocarrier systems containing polymers or co-delivered drugs may further enhance therapeutic efficiency [34]. In addition, the intrinsic magnetic properties of IONPs open opportunities for theranostic applications, such as MRI-guided delivery and hyperthermia-based therapies [21].

In conclusion, AG-IONPs demonstrate stability, biocompatibility, and multifunctionality, making them a promising nanoplatform for GBM therapy. While in vitro findings are encouraging, comprehensive in vivo studies addressing BBB penetration, pharmacokinetics, biodistribution, and therapeutic efficacy are required before clinical translation can be realized.

## 5. Conclusions

This study demonstrates that andrographolide-loaded iron oxide nanoparticles (AG-IONPs) can be successfully synthesized using a reverse co-precipitation method, yielding stable and biocompatible nanocarriers with favorable physicochemical properties for brain tumor targeting. The AG-IONPs exhibited high encapsulation efficiency, long-term stability, and potent in vitro anti-glioblastoma activity, as evidenced by dose- and time-dependent cytotoxicity and significant inhibition of cell migration. These findings suggest that integrating the natural anticancer properties of andrographolide with the delivery and imaging advantages of iron oxide nanoparticles offers a promising nanoplatform for glioblastoma therapy. Future studies should focus on evaluating blood–brain barrier penetration, pharmacokinetics, in vivo antitumor efficacy, and theranostic applications to support clinical translation.

## Figures and Tables

**Table 1:** Physicochemical characterization of nanoparticles.

Sample	DLS, *d^h^* (nm)	TEM (nm)	Zeta Potential (mV)
IONPs	2–12	7.0 ± 0.15	21.22 ± 1.58
AG-IONPs	2–25	13.5 ± 1.25	8.68 ± 0.87

**Table 2:** Half-maximal lethal concentration (LC_50_, µg/mL) values of andrographolide-loaded iron oxide nanoparticles (AG-IONPs) and temozolomide (TMZ) in DBTRG-05MG glioblastoma cells after 24, 48, and 72 h of treatment. AG-IONPs demonstrated a progressive reduction in LC_50_ values over time (44.01 ± 3.23 μM at 24 h to 15.82 ± 2.30 μM at 72 h), indicating increasing cytotoxic potency with longer exposure. TMZ was used as the positive control for comparison, confirming the strong antiproliferative activity of AG-IONPs against glioblastoma cells.

Time Frame	AGIONPs (µg/mL)	TMZ (µg/mL)
24 h	44.01 ± 3.23	19.46 ± 2.58
48 h	20.33 ± 3.02	15.06 ± 3.09
72 h	15.82 ± 2.30	11.80 ± 3.04

## Data Availability

The original contributions presented in this study are included in the article. Further inquiries can be directed to the corresponding author.

## Abbreviations

The following abbreviations are used in this manuscript:

AG	Andrographolide
ANOVA	Analysis of Variant
AT-FTIR	Attenuated Total Reflectance–Fourier Transform Infrared Spectroscopy
BBB	Blood–Brain Barrier
CO_2_	Carbon Dioxide
DBTRG-05MG	Human Glioblastoma multiforme cell line
DLS	Dynamic Light Scattering
DTS1070	Folded Capillary Cell for Zeta Potential Measurement (Malvern Panalytical)
EE	Encapsulation Efficiency
ERK 1/2	Extracellular Signal-Regulated Kinases 1 and 2
FBS	Fetal Bovine Serum
FTIR	Fourier Transform Infrared Spectroscopy
HCLE	Human Corneal Limbal Epithelial cell line
HDI	Human Development Index
HNO_3_	Nitric Acid
IC_50_	Half-maximal Inhibitory Concentration
INFORMM	Institute for Research in Molecular Medicine, Universiti Sains Malaysia
IO	Iron Oxide
IONPs	Iron Oxide Nanoparticles
IR	Infrared
LC_50_	Lethal Concentration for 50% of cells
LE	Loading Efficiency
MCF 7	Human breast adenocarcinoma cell lines
MDCK	Madin–Darby Canine Kidney cell line
MRI	Magnetic Resonance Imaging
OH	Hydroxyl group
PBS	Phosphate-Buffer Saline
PES	Polyethersulfone
PLGA	Poly(lactic-co-glycolic acid)
RPMI	Roswell Park Memorial Institute culture medium
TEM	Transmission Electron Microscope
TMZ	Temozolomide
USM	Universiti Sains Malaysia
UV	Ultraviolet
VIS	Visible Spectrum
WST-1	Water-Soluble Tetrazolium salt-1 assay
